# Long-Term Alcohol-Activated c-Jun N-terminal Kinase Isoform 2 Preserves Cardiac Function but Drives Ca^2+^-Triggered Arrhythmias

**DOI:** 10.3390/cells12182233

**Published:** 2023-09-08

**Authors:** Nikola Ricchiuti, Kurtis Chenoweth, Xianlong Gao, Dan J. Bare, Jiajie Yan, Xun Ai

**Affiliations:** Department of Physiology and Cell Biology, College of Medicine/Wexner Medical Center, The Ohio State University, 333 W 10th Avenue, Columbus, OH 43210, USA

**Keywords:** atrial fibrillation, stress kinase JNK2, Ca^2+^ signaling, cardiac function

## Abstract

Long-term alcohol consumption leads to cardiac arrhythmias including atrial fibrillation (AF), the most common alcohol-related arrhythmia. While AF significantly increases morbidity and mortality in patients, it takes years for an alcoholic individual undergoing an adaptive status with normal cardiac function to reach alcoholic cardiomyopathy. The underlying mechanism remains unclear to date. In this study, we assessed the functional role of JNK2 in long-term alcohol-evoked atrial arrhythmogenicity but preserved cardiac function. Wild-type (WT) mice and cardiac-specific JNK2dn mice (with an overexpression of inactive dominant negative (dn) JNK2) were treated with alcohol (2 g/kg daily for 2 months; 2 Mo). Confocal Ca^2+^ imaging in the intact mouse hearts showed that long-term alcohol prolonged intracellular Ca^2+^ transient decay, and increased pacing-induced Ca^2+^ waves, compared to that of sham controls, while cardiac-specific JNK2 inhibition in JNK2dn mice precluded alcohol-evoked Ca^2+^-triggered activities. Moreover, activated JNK2 enhances diastolic SR Ca^2+^ leak in 24 h and 48 h alcohol-exposed HL-1 atrial myocytes as well as HEK-RyR2 cells (inducible expression of human RyR2) with the overexpression of tGFP-tagged active JNK2-tGFP or inactive JNK2dn-tGFP. Meanwhile, the SR Ca^2+^ load and systolic Ca^2+^ transient amplitude were both increased in ventricular myocytes, along with the preserved cardiac function in 2 Mo alcohol-exposed mice. Moreover, the role of activated JNK2 in SR Ca^2+^ overload and enhanced transient amplitude was also confirmed in long-term alcohol-exposed HL-1 atrial myocytes. In conclusion, our findings suggest that long-term alcohol-activated JNK2 is a key driver in preserved cardiac function, but at the expense of enhanced cardiac arrhythmogenicity. Modulating JNK2 activity could be a novel anti-arrhythmia therapeutic strategy.

## 1. Introduction

Long-term alcohol consumption leads to cardiac arrhythmias, including both atrial and ventricular arrythmias, with atrial fibrillation (AF) being the most common alcohol-related arrhythmia; however, it can take years for an alcoholic individual undergoing adaptive cardiac changes, with normal function, to reach the point of alcoholic cardiomyopathy [1,2,3,4]. The underlying mechanism(s) remain unclear.

In the United States and South America, more than half of all women, and 60% to 80% of all men consume alcohol, while at least 50% of adults are active consumers of alcohol in Europe [5]. The Centers for Disease Control and Prevention (CDC) and other studies [5,6] reported that 61.2% of US adults are current drinkers, and 14% were former drinkers, and about 15 million Americans were classified as heavier drinkers (consuming > 7 drinks/week if female, and 14 drinks/week if male; 1 US standard drink = 14 g alcohol). During the COVID-19 pandemic, increased alcohol consumption, due to social isolation and enhanced mental stress, has magnified this serious alcohol abuse problem [7,8].

It is known that an excessive alcohol intake activates the stress-response kinase JNK, leading to organ injury [9,10,11,12,13,14], and long-term alcohol exposure alters intracellular Ca^2+^ homeostasis, which could underly enhanced arrhythmogenicity [15,16]. Our laboratory recently reported a causal link between the activated cardiac JNK isoform 2 and AF risk [17,18,19,20]. In the present study, we assessed the effect of alcohol exposure (2 g/kg for 2 months (2 Mo)) on JNK2 activation and on triggered abnormal Ca^2+^ activities in intact mouse atria, using high-resolution confocal Ca^2+^ imaging in Langendorff-perfused intact mouse hearts (i.e., with no neural regulatory inputs present). We have previously reported that cardiac diastolic sarcoplasmic reticulum (SR) Ca^2+^-handling dysfunction is a key abnormal ectopic trigger that initiates arrhythmias, and that this Ca^2+^ mishandling even causes cardiac pathological remodeling [18,20,21]. The functional role of JNK2 in SR Ca^2+^ handling was measured in cardiac myocytes freshly isolated from 2 Mo alcohol-exposed mice, and in a heterologous cellular model of HER-RyR2 cells with an inducible expression of human RyR2 channels and other SR Ca^2+^ handling proteins, as previously characterized [22]. Our findings suggest that the activation of atrial JNK2 is a key driver in long-term alcohol-exposure-evoked Ca^2+^-triggered arrhythmic activities, but also contributes to the maintenance of normal cardiac function. Modulating JNK2 activity could be a potential novel therapeutic strategy for alcoholic-related AF.

## 2. Methods

### 2.1. Animal and Cell Preparations

All animal studies followed the Guide for the Care and Use of Laboratory Animals (NIH Publication, 8th Edition, 2011), and were approved by the Institutional Animal Care and Use Committees of The Ohio State University, Rush University Medical Center, and Loyola University at Chicago. The terminal experiments were performed within 24 h following the conclusion of the alcohol course treatments. Wild-type (WT) C57B/6j male mice (2–2.5 months of age; The Jackson Laboratory, Bar Harbor, ME, USA) and cardiac-specific JNK2dn transgenic (Tg) mice with an overexpression of inactive JNK2 dominant-negative protein [18,20,23] were treated with alcohol (2 g/kg BW, intraperitoneal injection; i.p.) for a total of 2 months (2 Mo). Prior to the terminal studies, the sedated mice (2–3% isoflurane with inhaled oxygen) underwent echocardiographic examination, to acquire systolic and diastolic LV dimensional parameters from M-mode images, to calculate the ejection fraction (EF; calculated from the left ventricular systolic and diastolic dimensions). The mice were then sacrificed under a surgical plane of anesthesia induced with a mixture of 100 mg/kg ketamine and 4.5 mg/kg xylazine (i.p.) for either myocyte isolation, or intact heart Langendorff perfusion for confocal imaging studies. Cardiac myocytes were isolated from the Langendorff-prepared hearts undergoing enzyme (Liberase TH; Roche, Basel, Switzerland) digestion, as previously described [20,21].

A cultured HL-1 atrial myocyte line was used for our studies, as previously described [17,18,19,20,21]. The HL-1 cell line is a well-characterized mouse atrial myocyte line, expressing cardiac genes and proteins normally found in adult myocytes, as we and others have previously verified [17]. The HL-1 myocytes were strictly maintained and passaged as previously described, to ensure that they retained their normal morphological and electrophysiological qualities [1,2,17,24]. These HL-1 myocytes were then treated with alcohol (50 mM) for 24 h or 48 h, respectively. To test the effect of alcohol on the SR Ca^2+^ leak, the HL-1 myocytes were treated with 50 mM ethanol for 24 or 48 h with and without the presence of 170 nM JNK2 inhibitor JNK2I (EMD Millipore, Burlington, MA, USA), as previously described [18,19,20,21].

In addition, we used a well-characterized heterologous HEK cell line with doxycycline-inducible expression of human RyR2 and other SR Ca^2+^ load-handling proteins, as previously described [21,25]. HEK-RyR2 cells with transiently expressed turbo-GFP-tagged JNK2 (JNK2-tGFP) and JNK2dn-tGFP (dominant-negative) proteins were utilized for confocal cellular Ca^2+^ imaging. In brief, stable doxycycline-inducible HEK-293 cells with heterologous WT-RyR2 (RyR2-HEK-293) were cultured on coverslips, and RyR2 expression was induced with doxycycline (2 μg/mL). The plated cells were then transfected with either *JNK2-tGFP* (active human JNK2) or *JNK2dn-tGFP* (inactive human JNK2) mammalian expression vectors in 2 mL of DMEM, as previously described [21]. Following a 1.5 h incubation period, this medium was removed, the cells were gently washed twice with additional DMEM, and then complete DMEM-RyR2 medium (10% fetal bovine serum plus supplements), again containing doxycycline (2 μg/mL), was added for an overnight incubation. The following day, confocal Ca^2+^ imaging of identifiable tGFP-positive cells (green fluorescence; excitation/emission 482/502 nm) was conducted.

### 2.2. Biochemical Assays

The JNK2-isoform-specific activity was measured in 2 Mo alcohol-exposed mouse hearts versus sham controls from immunoprecipitating (IP) JNK2 protein, using anti-JNK2-specific antibodies (Ab76125: Abcam; Waltham, MA, USA), followed by measurement of the production of ADP in the kinase reaction with a JNK-specific enzyme substrate c-Jun, using an ADP-Glo^TM^ Kinase assay kit (Promega, Madison, WI; V6930). The reaction was performed using a kinase reaction buffer system (Tris·HCl 40 mM, MgCl_2_ 10 mM, *β*-glycerophosphate 5 mM, Na_3_VO_4_ 0.2 mM, pH 7.4), in the presence of 20 µM ATP for 45 min, as previously described [18,21,26].

### 2.3. Confocal Ca^2+^ Imaging on Intact Mouse Hearts

For the obtention of the whole mouse hearts for the confocal Ca^2+^ imaging studies, the mice were anesthetized using 2–4% isoflurane delivered with 100% oxygen, and were subsequently euthanized in a humane manner. The freshly harvested intact mouse hearts were cannulated, and subjected to Langendorff perfusion with oxygenated Ca^2+^-free Tyrode’s solution (137 mM NaCl, 0.36 mM NaH_2_PO_4_, 5.56 mM glucose, 1 mM MgCl_2_, 10 mM HEPES, 12 mM NaHCO_3_, pH 7.4) at room temperature, to eliminate blood in the coronary system. Subsequently, each heart was loaded with the Ca^2+^-sensitive dye Rhod2-AM (Invitrogen; Waltham, MA, USA) (5 μM in 10% Pluronic acid F-127 (AAT Bioquest; Sunnyvale, CA, USA) dissolved in DMSO) for 30 min. Following the dye staining, the intact heart was transferred to oxygenated 1.8 mM Ca^2+^ Tyrode’s perfusion at 37 °C. The imaging of Ca^2+^ transients in the intact mouse atria was performed using a confocal microscope (Nikon Eclipse TE2000-U, 40× objective, NA 1.3). The Rhod-2 dye was excited using an argon laser at 560 nm, and the emitted light was collected at a wavelength of 590 nm. The diastolic threshold was established as the minimum voltage required to achieve 1:1 atrial capture. Confocal line scans at 2 µs/pixel were conducted during sinus rhythm and after pacing, at frequencies of 8 Hz, 10 Hz, and 20 Hz.

The Ca^2+^ transient signals were normalized to the background level of fluorescence (ΔF/F_0_), where F_0_ represents the resting fluorescence during diastole in the same cardiac myocyte. The Ca^2+^ transient decay constant τ was calculated as the time required for the Ca^2+^ transient peak to decay to approximately 63.2% of its systolic peak value. Ca^2+^ waves were identified as abnormal Ca^2+^ release events from the RyR2 that were unsynchronized with normal sinus rhythm Ca^2+^ transients. The frequency of Ca^2+^ waves was quantified in terms of waves per millimeter per second. The intracellular Ca^2+^ transient decay constant τ and Ca^2+^ wave frequency were analyzed using a well-established customized MATLAB-based algorithm.

### 2.4. Cellular Confocal Ca^2+^ Imaging in Myocytes and Heterologous HEK-RyR2 Cells

Cultured HL-1 mouse atrial myocytes, freshly isolated mouse ventricular myocytes, and transfected HEK-RyR2 cells were used for cellular confocal Ca^2+^ imaging studies. Prior to the imaging, the cells were loaded with Fluo-4 AM dye (4.6 µM, in normal Hanks Salt solution, Invitrogen; Waltham, MA, USA) for 15 min at 34.5 °C, followed immediately by confocal Ca^2+^ imaging studies (Nikon Eclipse TE2000-U, 40× objective, NA 1.3). During imaging, the myocytes were continuously perfused with normal Hanks’ salt solution at 35 °C, and physiological pH (NaCl 136 mM, NaHCO_3_ 4.16 mM, HEPES 5.04 mM, KCl 5.36 mM, KH_2_PO_4_ 0.44 mM, NaH_2_PO_4_ 0.4 mM, MgSO_4_ 0.81 mM, CaCl_2_ 1.26 mM, glucose 5.05 mM, pH 7.4). The Fluo-4 AM dye was excited with an argon laser at a wavelength of 488 nm, and the emitted light was collected at a wavelength of 515 nm.

To measure the SR Ca^2+^ leak, the myocytes were superfused with 0 Na^+^/0 Ca^2+^ Tyrode’s solution (140 mM LiCl, 4 mM KCl, 1 mM MgCl_2_, 4 mM HEPES, 10 mM EGTA, pH 7.4) for 10 min. A pre-tetracaine confocal recording was taken, after which the cells were superfused with tetracaine (1 mM, dissolved in 0 Na^+^/0 Ca^2+^ Tyrode’s), and a post-tetracaine recording was taken. The Ca^2+^ leak was calculated as the intracellular Ca^2+^ fluorescence decline following the superfusion of the cells with tetracaine. The rapid decline in fluorescence signal after the tetracaine exposure represents the diastolic SR Ca^2+^ that “leaked” through the RyR2 channel, given that tetracaine rapidly lowers the RyR2 open probability with blocked NCX activity via the 0 Na^+^/0 Ca^2+^ Tyrode’s solution. The fluorescence readings were defined as the collected steady-state fluorescence readings at the beginning of the recording (F_0_). The Ca^2+^ transient signals were reported as ΔF/F_0_, which is the change in fluorescence normalized to the baseline (F_0_) readings.

To measure the SR Ca^2+^ load, the myocytes were, instead, superfused with 1.8 mM Ca^2+^ Tyrode’s solution at room temperature (137 mM NaCl, 0.36 mM NaH_2_PO_4_, 5.56 mM glucose, 1 mM MgCl_2_, 10 mM HEPES, 12 mM NaHCO_3_, pH 7.4), and a baseline recording was taken. Then, the monocyte layer was superfused with caffeine (0.67 mM, dissolved in 1.8 mM Ca^2+^ Tyrode’s), to obtain a post-caffeine image. The Ca^2+^ transient amplitude was defined as the peak values of the Ca^2+^ transients against the diastolic background (F_0_). The SR Ca^2+^ leak, load, and transient amplitude were all analyzed using a well-established customized MATLAB (Natick, MA, USA)-based algorithm.

### 2.5. Statistical Analyses

All data are presented as mean ± SEM. The differences between multiple groups or any two groups were evaluated using one-way ANOVA with the post-hoc Tukey test or Student’s *t*-test. When a heterogeneity of variance was observed, a nonparametric Mann–Whitney *U* test or nonparametric one-way ANOVA was performed. A *p*-value of <0.05 was considered significant.

## 3. Results

### 3.1. Long-Term Alcohol Exposure Activates Atrial JNK2 and Increases Ca^2+^-Triggered Arrhythmic Activities in Intact Atria

**Figure 1:**
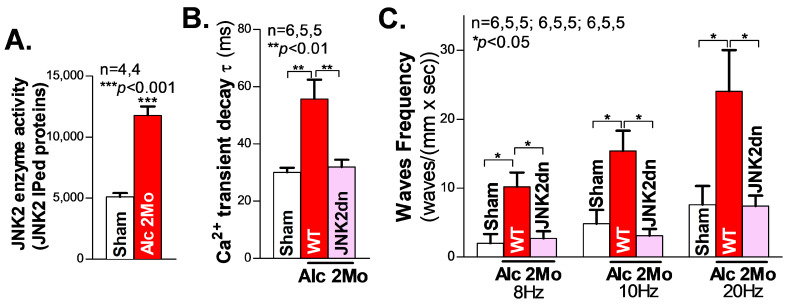
Activated JNK2 drives alcohol-induced Ca^2+^ mishandling in long-term alcohol-treated atria. (**A**) The in vitro kinase activity assay shows that the immunoprecipitated JNK2 (IPed: JNK2) kinase activity is significantly elevated in the hearts of mice exposed to 2 Mo alcohol, compared to the sham controls. (**B**) The summarized data from high-resolution confocal Ca^2+^ imaging, showing a significantly increased Ca^2+^ transient decay constant τ in 2 Mo alcohol-exposed wildtype (WT) mice, but not in cardiac-specific JNK2-inhibited JNK2dn mice, compared to the sham controls. (**C**) The 2 Mo alcohol-treated WT mouse hearts had significantly increased electrical pacing-induced aberrant Ca^2+^ waves at 8 Hz, 10 Hz, and 20 Hz, compared to the non-alcohol sham controls, while these alcohol-evoked events were effectively precluded in the JNK2dn mice.

We first assessed the cardiac JNK2 activity in 2-month (2 Mo) alcohol-exposed mouse hearts by immunoprecipitating (IP) the JNK2 proteins with a JNK2-isoform-specific antibody, followed by JNK2 enzyme activity measurement (reflected in the ADP production), using the ADP-Glo kinase assay kit (Promega; Madsion, WI, USA), as we previously described [18,26]. In the 2 Mo alcohol-exposed WT mouse hearts, there was a 1.2-fold increase in JNK2 activity, compared to the sham controls (Figure 1A).

We and others have previously shown that abnormal Ca^2+^ triggers are critical in generating abnormal ectopic activities and enhanced arrhythmogenesis in heart failure and AF [18,20,27,28,29]. To elucidate the role of JNK2 in alcohol-induced Ca^2+^-triggered activities, high-resolution confocal Ca^2+^ imaging was conducted on Langendorff-perfused intact mouse hearts. We firstly recorded line-scan confocal Ca^2+^ transient images of intact atria (pre-loaded with a Ca^2+^ indicator, Rhod2-AM) during their intrinsic sinus rhythm. Figure 1B shows the summarized data demonstrating how the atrial Ca^2+^ transient decay constant τ was prolonged in 2 Mo alcohol-exposed WT mice compared to sham WT mice (55.9 ± 2.74 ms vs. 30.03 ± 6.72 ms in WT sham), reflecting the elevated level of diastolic intracellular Ca^2+^, and the increased risk of generating arrhythmic activities. Next, hearts were electrically paced, and then the pacing was stopped, to permit the inherent sinus rhythm to resume. The summarized data in Figure 1C show that 2 Mo alcohol-exposed WT mouse atria exhibited a significantly increased frequency of arrhythmic Ca^2+^ waves compared to sham controls following 8 Hz (10.20 ± 2.07 waves/(mm × s) vs. 1.99 ± 1.33 waves/(mm × s) in the sham), 10 Hz (15.38 ± 2.94 waves/(mm × s) vs. 4.84 ± 1.99 waves/(mm × s) in the WT sham), and 20 Hz (24.05 ± 5.98 waves/(mm × s) vs. 7.59 ± 2.72 waves/(mm × s)) electrical pacing. Thus, long-term alcohol exposure enhances Ca^2+^-triggered atrial arrhythmogenicity.

JNK activity is known to be reflected by the phosphorylation status of the amino acid residues of Threonine 183 and Tyrosine 185 on the “TPY motif” (JNK-P) [30,31]. To specifically assess the role of alcohol-activated JNK2 in this increased arrythmia risk, a unique JNK2dn transgenic mouse model with a cardiac-specific overexpression of inactive JNK2 dominant-negative (JNK2dn) proteins with mutations of T183A and Y185F, which competitively inhibits endogenous JNK2, was exposed to alcohol for 2 months. Strikingly, the overexpression of inactive JNK2dn proteins eliminated the alcohol-triggered Ca^2+^ waves following 8 Hz (10.20 ± 2.07 waves/(mm × s) in 2 Mo Alc-WT vs. 2.71 ± 1.03 waves/(mm × s) in JNK2dn-Alc), 10 Hz (15.38 ± 2.94 waves/(mm × s) in WT Alc vs. 3.11 ± 0.96 waves/(mm × s) in JNK2dn-Alc), and 20 Hz (24.05 ± 5.98 waves/(mm × s) in WT-Alc vs. 7.34 ± 1.53 waves/(mm × s) in JNK2dn-Alc) burst pacing (Figure 1C). Similarly, alcohol-evoked transient τ prolongation was also normalized in the JNK2dn mice compared to the controls (Figure 1B). Taken together, our findings suggest that long-term alcohol-activated JNK2 drives Ca^2+^-triggered arrhythmic activities in intact atria.

### 3.2. Activated JNK2 Drives Aberrant Diastolic SR Ca^2+^ Leak

**Figure 2:**
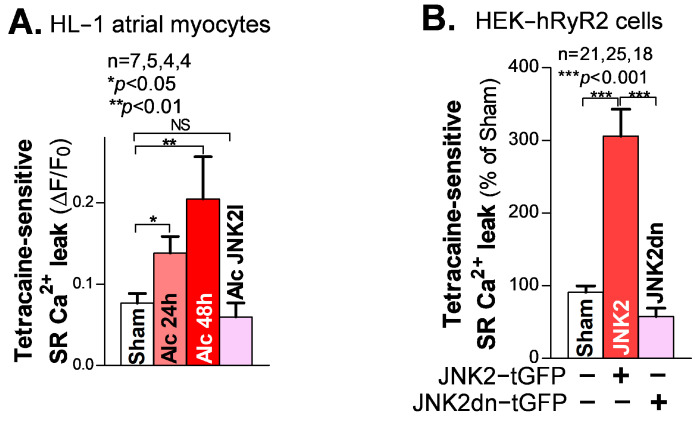
Activated JNK2 increases diastolic SR Ca^2+^ leak. (**A**) The summarized data suggest that the alcohol-evoked tetracaine-sensitive SR Ca^2+^ leak is significantly increased in 24 h and 48 h alcohol-treated HL-1 atrial myocytes (37°C), and increases in a time-dependent fashion, compared to sham controls. Strikingly, pharmacological JNK2 inhibition (JNK2I, far-right bar) eliminated alcohol (24 h)-induced SR Ca^2+^ leak, effectively blocking the evoked leak to wildtype sham levels. (**B**) The summarized data showing increased tetracaine-sensitive diastolic SR Ca^2+^ leak in JNK2-tGFP-vector-transfected HEK-hRyR2 cells, compared to sham controls (left bar) and inactive JNK2dn-tGFP-vector-transfected cells. NS denotes ‘not significant’.

SR Ca^2+^ mishandling is a crucial factor in Ca^2+^-triggered arrhythmic events, with enhanced diastolic SR Ca^2+^ leak leading to increased abnormal Ca^2+^ waves. Here, we used our well-validated tetracaine-sensitive SR Ca^2+^ leak measurement under confocal microscopy, as previously described [18,20,21]. We found that following 24 h and 48 h alcohol exposure, HL-1 atrial myocytes showed a time-dependent increase in the level of tetracaine-sensitive SR Ca^2+^ leak, compared to the sham controls (Figure 2A). Similar to the rescue effect of JNK2-specific inhibition in the intact atria on triggered Ca^2+^ waves, as shown in Figure 1C, this alcohol-evoked leak was precluded when a JNK2-specific inhibitor (JNK2-I; 170 nM) was present (Figure 2A), suggesting a causative role of activated JNK2 in this long-term alcohol-evoked aberrant diastolic SR Ca^2+^ leak.

To further determine the specific action of activated JNK2 in diastolic SR Ca^2+^ leak, we applied a previously well-characterized heterologous HEK-RyR2 cell line, with the doxycycline-induced expression of cardiac RyR2 and other SR Ca^2+^ load-handling proteins, as previously described [21,25]. We transfected HEK-RyR2 cells with turbo-GFP (tGFP)-tagged JNK2 vectors, followed by measurement of the tetracaine-sensitive SR Ca^2+^ leak in tGFP-positively expressing HEK-RyR2 cells, using a confocal cellular Ca^2+^ imaging technique. Figure 2B shows the summarized data of the significantly increased tetracaine-sensitive SR Ca^2+^ leak in active JNK2-tGFP-positively expressing HEK-RyR2 cells (green cells), compared to sham controls. To further assess whether the JNK2 activity is a key factor in its function in SR Ca^2+^ leak, we transfected HEK-RyR2 cells with a JNK2dn vector, to express inactive JNK2dn proteins. Figure 2B (far-right bar) shows that inactive JNK2dn-tGFP-expressing HEK-RyR2 cells exhibit a normal level of Ca^2+^ leak, seen in sham controls, suggesting that JNK2 is responsible for the abnormal diastolic SR Ca^2+^ leak.

### 3.3. Long-Term Alcohol-Exposure-Activated JNK2 with Augmented SR Ca^2+^ Load and Maintained Normal Cardiac Function

**Figure 3:**
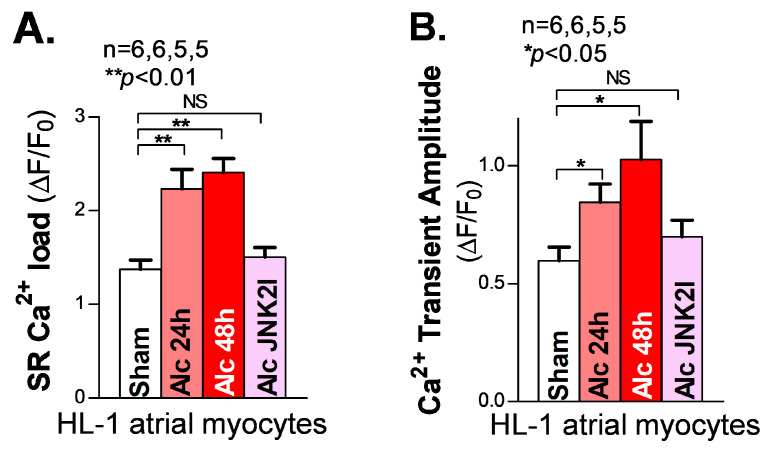
Activated JNK2 elevates the SR Ca^2+^ load and systolic Ca^2+^ transient amplitude in cultured HL-1 atrial myocytes. (**A**,**B**) The summarized data showing an increased SR Ca^2+^ load (**A**) and Ca^2+^ transient amplitude (**B**), measured from cellular confocal Ca^2+^ images in 24 h and 48 h alcohol-treated (two middle bars) HL-1 atrial myocytes, while JNK2 inhibition precluded this alcohol-increased SR Ca^2+^ load and Ca^2+^ transient amplitude (far-right bars). NS denotes ‘not significant’.

A sufficient diastolic SR Ca^2+^ load is essential to supplying enough systolic Ca^2+^ released from the SR to generate the normal systolic Ca^2+^ transients required for normal contractions of the cardiac muscles. In the myocyte relaxation phase, the SR Ca^2+^ release almost completely shuts off (∼99%), and most of the remaining cytosolic Ca^2+^ is pumped back into the SR by the Ca^2+^ pump (SERCA2) [32]. A reduced SR load is known to be a hallmark of reduced cardiac function in heart failure patients [27,33]. However, our recently reported findings [18,21] of JNK2-augmented SR Ca^2+^ load via enhanced SERCA2 pump activity suggest that JNK2 could play a key role in maintaining the normal cardiac output as an adaptive mechanism in response to binge alcohol exposure [18,21]. Here, we assessed the SR Ca^2+^ load in long-term alcohol-exposed HL-1 myocytes. We found a significantly increased SR Ca^2+^ load by measuring the peak of the 10 mM caffeine-induced Ca^2+^ transients in both 24 h and 48 h alcohol-exposed HL-1 atrial myocytes, compared to sham controls (Figure 3A, left vs. middle two bars). Again, JNK2-specific inhibition by JNK2I strikingly eliminated the alcohol-evoked elevation of the SR Ca^2+^ load (Figure 3A, far-right bar). Moreover, Figure 3B shows a gradually increased systolic intracellular Ca^2+^ transient amplitude in 24 h and 48 h alcohol-exposed cultured HL-1 myocytes, respectively, while JNK2-specific inhibition mitigated these long-term alcohol-exposure-caused effects on the SR Ca^2+^ load and transient amplitude, compared to the sham controls.

**Figure 4:**
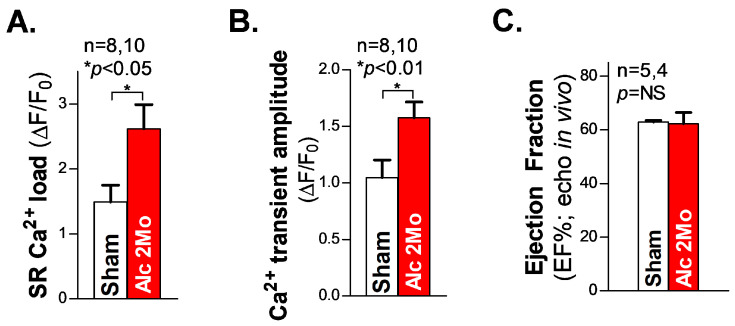
The increased SR Ca^2+^ load and Ca^2+^ transient amplitude in ventricular myocytes with preserved cardiac function in 2 Mo alcohol-exposed mice. (**A**,**B**) The summarized data of the increased SR Ca^2+^ load (**A**) and Ca^2+^ transient amplitude (**B**) in freshly isolated ventricular myocytes from 2 Mo alcohol-exposed mice, compared to the sham controls. (**C**) The pooled data of echocardiography showing an unchanged ejection fraction (EF) in 2 Mo alcohol-exposed mice, compared to sham controls. NS denotes ‘not significant’.

Next, we measured the SR Ca^2+^ load in freshly isolated ventricular myocytes from 2 Mo alcohol-exposed mice. The summarized cellular confocal Ca^2+^ data reveal that there is significantly elevated SR Ca^2+^ load (Figure 4A), along with an increased systolic Ca^2+^ transient amplitude (Figure 4B), in 2 Mo alcohol-exposed ventricular myocytes, compared to 2 Mo saline-treated sham controls. We also assessed the cardiac function in living mice using echocardiography measurements. The pooled data of the ejection fraction (EF) in 2 Mo alcohol-exposed WT mice showed comparable values to those of the sham controls (Figure 4C), reflecting a maintained normal cardiac function in 2 Mo alcohol-exposed mice. We can infer that JNK2 plays a key role in maintaining normal cardiac function in long-term alcohol-exposed hearts, through an adaptive mechanism of the JNK2-driven SR Ca^2+^ overload necessary to compensate for the loss in SR Ca^2+^ content resulting from a greater diastolic SR Ca^2+^ leak.

## 4. Discussion

Our current study has revealed a compelling causal role for JNK2 activation in long-term alcohol-exposure-evoked atrial arrhythmogenicity with maintained cardiac function, through the JNK2-specific actions of an enhanced aberrant diastolic SR Ca^2+^ leak, accompanied by an augmented SR Ca^2+^ load. These findings are significant because they highlight the dual action of long-term alcohol-activated JNK2 in altered myocyte Ca^2+^ homoeostasis, which may be able to explain a long-time unresolved mystery occurring in individuals with a long history of excessive alcohol consumption that have a high risk of developing cardiac arrhythmias, but can maintain a normal level of cardiac function for many years.

Long-term excessive alcohol consumption leads to a significant increase in cardiac arrhythmias, and AF is the most common arrhythmia type, with a dramatically increased risk of mortality and morbidity, and adds vast economic costs to healthcare spending [1,2,3,4,34,35,36,37,38]. According to the CDC, an estimated 12.1 million people in the United States are expected to have AF by 2030, which will lead to a tremendous burden on our healthcare system and society. Recent studies suggested a significant dose- and time-dependent effect of alcohol consumption on AF genesis, and the high AF risk is also associated with long-term alcohol abuse, even in those without co-existing cardiovascular diseases [39,40,41,42]. Unfortunately, the success rate of rehabilitation from chronic alcohol abuse for those wishing to stop drinking is low, at between 5% and 30% [43,44]. Although AF is a growing public health concern, the currently available treatment strategies, including anti-arrhythmic drugs and atrial ablation, for alcohol-related AF remain suboptimal, largely due to the lack of understanding of the underlying mechanisms. Thus, the exploration of alternative effective pharmacological targets is clearly needed, to improve therapeutic effectiveness and reduce costs for AF patients and our healthcare system.

Our findings for the JNK2-specific actions in [Ca^2+^]_SR_ mishandling shed new light on modulating JNK2 activity as a promising anti-AF therapeutic strategy. Accumulating evidence suggests that (acute or chronic) alcohol-driven JNK activation seems to be common, and contributes to alcohol-related cell death and tissue injury [9,10,11,12,13,29,45]. In the heart, JNK1 and JNK2 are the major isoforms, while JNK3 is expressed at a much lower level [21,46]. To date, the function of JNK2 has received much less attention [17,47,48]. Our group is the first to report the JNK2-specific action on enhanced atrial arrhythmogenesis in aged and binge-alcohol-exposed hearts. Here, we provide new evidence demonstrating the key role of activated JNK2 in long-term alcohol-evoked diastolic SR Ca^2+^ leak and triggered arrhythmias. Further, this long-term alcohol-activated JNK2 is also essential in maintaining cardiac contractile function, through the JNK2-specific action in augmented SR Ca^2+^ load for a sufficient Ca^2+^ transient amplitude, in order to maintain a normal cardiac output.

Dysfunctional SR Ca^2+^ handling is known to play a key role in cardiac adaptive and maladaptive remodeling during the development of heart failure [27,45,49,50]. Under physiological conditions, systolic Ca^2+^-triggered SR Ca^2+^ release produces a large intracellular Ca^2+^ transient that drives cell contraction, while Ca^2+^ release stops during the diastolic phase, and excess cytosolic Ca^2+^ ions are removed from the cytosol, either through pumping Ca^2+^ back to SR (via the SERCA2 function), or extruding Ca^2+^ from the cell [2]. Under pathological conditions, an abnormally high SR Ca^2+^ leak will reduce the SR Ca^2+^ load and, consequently, reduce the systolic fractional SR Ca^2+^ release for a given L-type voltage-gated Ca^2+^ current (I_Ca_) trigger, consequently reducing the systolic Ca^2+^ transient amplitude, as commonly seen in the failing heart [27,29,45]. Propagating Ca^2+^ waves can be triggered by an excessive diastolic NCX-mediated influx from elevated intracellular Ca^2+^, which may produce abnormal triggered activities (e.g., DADs), and initiate arrhythmias [32,45]. Our group recently reported, for the first time, that JNK2 specifically enhanced atrial arrhythmic Ca^2+^ events and AF susceptibility via an enhanced diastolic SR Ca^2+^ leak, while this JNK2-driven SR Ca^2+^ leak was associated with an elevated SR Ca^2+^ load in both aged and binge-alcohol-exposed hearts [18,20]. This result appears to contradict the more classic concept that a higher SR Ca^2+^ leak depletes the SR load that occurs in heart failure myocytes [27,45]. However, we further discovered that activated JNK2 directly enhances the SERCA2 activity, which explains the dichotomy as to how the systolic Ca^2+^ transient amplitude in both the atria and ventricles are unaffected by JNK2 activation induced by aging or binge alcohol exposure [18,20,21]. In the current study, our results clearly demonstrate that the JNK2-driven SERCA2 pump stimulation is sufficient to elevate the diastolic SR Ca^2+^ load in order to maintain a normal Ca^2+^ transient amplitude, despite a greater diastolic leak concordantly existing in long-term alcohol-exposed myocytes. In other words, long-term alcohol-activated JNK2, as a stress-induced regulator, maintains a high level of SR Ca^2+^ load in order to preserve cardiac function but, on the other hand, causes an enhanced arrhythmogenicity, due to diastolic SR Ca^2+^ leak. Thus, the functional outcome of the long-term activation of JNK2 could explain how an individual with a long history of excessive alcohol consumption has a significantly increased risk of cardiac arrhythmias, and yet their cardiac function remains normal for years.

It is known that abnormal activities in cells in the pulmonary veins have been shown to underlie the initiation of AF; the maintenance of AF is believed to be due to an arrhythmogenic substrate characterized by abnormal impulse propagation. This substrate includes structural (e.g., fibrosis) and molecular (e.g., gap junctions and other ion channels) remodeling [51,52]. We recently reported that JNK-associated AF is promoted by gap-junction remodeling, which contributes to impaired cell-to-cell communication, slows atrial AP conduction, and fosters reentry circuit formation in the absence of structural remodeling (i.e., fibrosis) [17,19]. In other words, JNK-impaired cell-to-cell communication fosters the maintenance of abnormal Ca^2+^-triggered ectopic triggers, to promote cardiac arrhythmia onset. While modulating JNK2 activity could be a promising approach to stopping Ca^2+^-triggered activity, and improving myocardial conduction and, consequently, eliminating AF in chronic alcohol users, further investigation is clearly warranted, to understand whether the inhibition of JNK2 would also allow the maintenance of abnormal cardiac function in long-term alcohol-exposed hearts. Exploring new drugs targeting JNK2 activity as a novel anti-arrhythmia therapeutic strategy for patients with an issue of long-term alcohol intake could be a future research direction.

## Data Availability

Raw and analyzed data used in this manuscript can be found in the link following https://www.dropbox.com/scl/fo/giq1tovh346g1i2qkr3t6/h?rlkey=o95t84vanzwtbh4ef7c504elq&dl=0 (accessed on 10 August 2023).

## Abbreviations

AF	atrial fibrillation
Ca^2+^	calcium
CDC	Centers for Disease Control and Prevention
dn	dominant negative
i.p.	intraperitoneal injection
IP(IPed)	immunoprecipitation, immunoprecipitated
JNK	c-Jun N-terminal kinase
JNK2dn	JNK2 dominant-negative protein
JNK2I	JNK2 inhibitor
LV	left ventricle
RyR	ryanodine receptor
SR	sarcoplasmic reticulum
WT	wild-type
Mo	month-old

## References

[1] Miyasaka Y., Barnes M.E., Gersh B.J., Cha S.S., Bailey K.R., Abhayaratna W.P., Seward J.B., Tsang T.S. (2006). Secular trends in incidence of atrial fibrillation in Olmsted County, Minnesota, 1980 to 2000, and implications on the projections for future prevalence. Circulation.

[2] Go A.S., Hylek E.M., Phillips K.A., Chang Y., Henault L.E., Selby J.V., Singer D.E. (2001). Prevalence of diagnosed atrial fibrillation in adults: National implications for rhythm management and stroke prevention: The AnTicoagulation and Risk Factors in Atrial Fibrillation (ATRIA) Study. JAMA.

[3] Rich M.W. (2009). Epidemiology of atrial fibrillation. J. Interv. Card. Electrophysiol..

[4] Lowenstein S.R., Gabow P.A., Cramer J., Oliva P.B., Ratner K. (1983). The role of alcohol in new-onset atrial fibrillation. Arch. Intern. Med..

[5] Griswold M.G., Fullman N., Hawley C., Arian N., Zimsen S.R., Tymeson H.D., Venkateswaran V., Tapp A.D., Forouzanfar M.H., Salama J.S. (2018). Alcohol use and burden for 195 countries and territories, 1990–2016: A systematic analysis for the Global Burden of Disease Study 2016. Lancet.

[6] Rehm J., Shield K.D. (2019). Global Burden of Alcohol Use Disorders and Alcohol Liver Disease. Biomedicines.

[7] Calina D., Hartung T., Mardare I., Mitroi M., Poulas K., Tsatsakis A., Rogoveanu I., Docea A.O. (2021). COVID-19 pandemic and alcohol consumption: Impacts and interconnections. Toxicol. Rep..

[8] Clay J.M., Parker M.O. (2020). Alcohol use and misuse during the COVID-19 pandemic: A potential public health crisis?. Lancet Public. Health.

[9] Endo J., Sano M., Katayama T., Hishiki T., Shinmura K., Morizane S., Matsuhashi T., Katsumata Y., Zhang Y., Ito H. (2009). Metabolic remodeling induced by mitochondrial aldehyde stress stimulates tolerance to oxidative stress in the heart. Circ. Res..

[10] Walker R.K., Cousins V.M., Umoh N.A., Jeffress M.A., Taghipour D., Al-Rubaiee M., Haddad G.E. (2013). The good, the bad, and the ugly with alcohol use and abuse on the heart. Alcohol. Clin. Exp. Res..

[11] Li S.Y., Gilbert S.A., Li Q., Ren J. (2009). Aldehyde dehydrogenase-2 (ALDH2) ameliorates chronic alcohol ingestion-induced myocardial insulin resistance and endoplasmic reticulum stress. J. Mol. Cell. Cardiol..

[12] Yang L., Wu D., Wang X., Cederbaum A.I. (2012). Cytochrome P4502E1, oxidative stress, JNK, and autophagy in acute alcohol-induced fatty liver. Free Radic. Biol. Med..

[13] Aroor A.R., Shukla S.D. (2004). MAP kinase signaling in diverse effects of ethanol. Life Sci..

[14] El-Mas M.M., Fan M., Abdel-Rahman A.A. (2013). Role of rostral ventrolateral medullary ERK/JNK/p38 MAPK signaling in the pressor effects of ethanol and its oxidative product acetaldehyde. Alcohol. Clin. Exp. Res..

[15] Lazarevic A.M., Nakatani S., Neskovic A.N., Marinkovic J., Yasumura Y., Stojicic D., Miyatake K., Bojic M., Popovic A.D. (2000). Early changes in left ventricular function in chronic asymptomatic alcoholics: Relation to the duration of heavy drinking. J. Am. Coll. Cardiol..

[16] Aistrup G.L., Kelly J.E., Piano M.R., Wasserstrom J.A. (2006). Biphasic changes in cardiac excitation-contraction coupling early in chronic alcohol exposure. Am. J. Physiol. Heart Circ. Physiol..

[17] Yan J., Kong W., Zhang Q., Beyer E.C., Walcott G., Fast V.G., Ai X. (2013). c-Jun N-terminal kinase activation contributes to reduced connexin43 and development of atrial arrhythmias. Cardiovasc. Res..

[18] Yan J., Thomson J.K., Zhao W., Gao X., Huang F., Chen B., Liang Q., Song L.S., Fill M., Ai X. (2018). Role of Stress Kinase JNK in Binge Alcohol-Evoked Atrial Arrhythmia. J. Am. Coll. Cardiol..

[19] Yan J., Thomson J.K., Zhao W., Wu X., Gao X., DeMarco D., Kong W., Tong M., Sun J., Bakhos M. (2018). The stress kinase JNK regulates gap junction Cx43 gene expression and promotes atrial fibrillation in the aged heart. J. Mol. Cell. Cardiol..

[20] Yan J., Zhao W., Thomson J.K., Gao X., DeMarco D.M., Carrillo E., Chen B., Wu X., Ginsburg K.S., Bakhos M. (2018). Stress Signaling JNK2 Crosstalk with CaMKII Underlies Enhanced Atrial Arrhythmogenesis. Circ. Res..

[21] Yan J., Bare D.J., DeSantiago J., Zhao W., Mei Y., Chen Z., Ginsburg K., Solaro R.J., Wolska B.M., Bers D.M. (2021). JNK2, a Newly-Identified SERCA2 Enhancer, Augments an Arrhythmic [Ca^2+^]SR Leak-Load Relationship. Circ. Res..

[22] Chen W., Wang R., Chen B., Zhong X., Kong H., Bai Y., Zhou Q., Xie C., Zhang J., Guo A. (2014). The ryanodine receptor store-sensing gate controls Ca^2+^ waves and Ca^2+^-triggered arrhythmias. Nat. Med..

[23] Petrich B.G., Molkentin J.D., Wang Y. (2003). Temporal activation of c-Jun N-terminal kinase in adult transgenic heart via cre-loxP-mediated DNA recombination. FASEB J..

[24] Yan J., Thomson J.K., Zhao W., Fast V.G., Ye T., Ai X. (2015). Voltage and calcium dual channel optical mapping of cultured HL-1 atrial myocyte monolayer. J. Vis. Exp..

[25] Kong H., Jones P.P., Koop A., Zhang L., Duff H.J., Chen S.R. (2008). Caffeine induces Ca^2+^ release by reducing the threshold for luminal Ca^2+^ activation of the ryanodine receptor. Biochem. J..

[26] Gao X., Wu X., Yan J., Zhang J., Zhao W., DeMarco D., Zhang Y., Bakhos M., Mignery G., Sun J. (2018). Transcriptional regulation of stress kinase JNK2 in pro-arrhythmic CaMKIIdelta expression in the aged atrium. Cardiovasc. Res..

[27] Ai X., Curran J.W., Shannon T.R., Bers D.M., Pogwizd S.M. (2005). Ca^2+^/calmodulin-dependent protein kinase modulates cardiac ryanodine receptor phosphorylation and sarcoplasmic reticulum Ca^2+^ leak in heart failure. Circ. Res..

[28] Yeh Y.H., Wakili R., Qi X.Y., Chartier D., Boknik P., Kääb S., Ravens U., Coutu P., Dobrev D., Nattel S. (2008). Calcium-handling abnormalities underlying atrial arrhythmogenesis and contractile dysfunction in dogs with congestive heart failure. Circ. Arrhythm. Electrophysiol..

[29] Respress J.L., van Oort R.J., Li N., Rolim N., Dixit S.S., deAlmeida A., Voigt N., Lawrence W.S., Skapura D.G., Skardal K. (2012). Role of RyR2 phosphorylation at S2814 during heart failure progression. Circ. Res..

[30] Kyriakis J.M., Avruch J. (1990). pp54 microtubule-associated protein 2 kinase. A novel serine/threonine protein kinase regulated by phosphorylation and stimulated by poly-L-lysine. J. Biol. Chem..

[31] Kyriakis J.M., Brautigan D.L., Ingebritsen T.S., Avruch J. (1991). pp54 microtubule-associated protein-2 kinase requires both tyrosine and serine/threonine phosphorylation for activity. J. Biol. Chem..

[32] Bers D.M. (2000). Calcium fluxes involved in control of cardiac myocyte contraction. Circ. Res..

[33] del Monte F., Harding S.E., Schmidt U., Matsui T., Kang Z.B., Dec G.W., Gwathmey J.K., Rosenzweig A., Hajjar R.J. (1999). Restoration of contractile function in isolated cardiomyocytes from failing human hearts by gene transfer of SERCA2a. Circulation.

[34] Benjamin E.J., Levy D., Vaziri S.M., D’Agostino R.B., Belanger A.J., Wolf P.A. (1994). Independent risk factors for atrial fibrillation in a population-based cohort. The Framingham Heart Study. JAMA.

[35] Benjamin E.J., Wolf P.A., D’Agostino R.B., Silbershatz H., Kannel W.B., Levy D. (1998). Impact of atrial fibrillation on the risk of death: The Framingham Heart Study. Circulation.

[36] Voskoboinik A., Prabhu S., Ling L.H., Kalman J.M., Kistler P.M. (2016). Alcohol and Atrial Fibrillation: A Sobering Review. J. Am. Coll. Cardiol..

[37] Di Salvo T.G., Winterfield J. (2018). Holiday Heart: Some Sobering Mechanistic Insights. J. Am. Coll. Cardiol..

[38] Balbao C.E., de Paola A.A., Fenelon G. (2009). Effects of alcohol on atrial fibrillation: Myths and truths. Ther. Adv. Cardiovasc. Dis..

[39] Djousse L., Levy D., Benjamin E.J., Blease S.J., Russ A., Larson M.G., Massaro J.M., D’Agostino R.B., Wolf P.A., Ellison R.C. (2004). Long-term alcohol consumption and the risk of atrial fibrillation in the Framingham Study. Am. J. Cardiol..

[40] Larsson S.C., Drca N., Wolk A. (2014). Alcohol consumption and risk of atrial fibrillation: A prospective study and dose-response meta-analysis. J. Am. Coll. Cardiol..

[41] Kodama S., Saito K., Tanaka S., Horikawa C., Saito A., Heianza Y., Anasako Y., Nishigaki Y., Yachi Y., Iida K.T. (2011). Alcohol consumption and risk of atrial fibrillation: A meta-analysis. J. Am. Coll. Cardiol..

[42] Samokhvalov A.V., Irving H.M., Rehm J. (2010). Alcohol consumption as a risk factor for atrial fibrillation: A systematic review and meta-analysis. Eur. J. Cardiovasc. Prev. Rehabil..

[43] Walsh D.C., Hingson R.W., Merrigan D.M., Levenson S.M., Cupples L.A., Heeren T., Coffman G.A., Becker C.A., Barker T.A., Hamilton S.K. (1991). A randomized trial of treatment options for alcohol-abusing workers. N. Engl. J. Med..

[44] Dodes L., Dodes Z. (2014). The Sober Truth: Debunking the Bad Science behind 12-Step Programs and the Rehab Industry.

[45] Bers D.M. (2014). Cardiac sarcoplasmic reticulum calcium leak: Basis and roles in cardiac dysfunction. Annu. Rev. Physiol..

[46] Kuan C.Y., Yang D.D., Samanta Roy D.R., Davis R.J., Rakic P., Flavell R.A. (1999). The Jnk1 and Jnk2 protein kinases are required for regional specific apoptosis during early brain development. Neuron.

[47] Rose B.A., Force T., Wang Y., van der Velden J., Stienen G.J.M., You J., Wu J., Zhang Q., Ye Y., Wang S. (2010). Mitogen-activated protein kinase signaling in the heart: Angels versus demons in a heart-breaking tale. Physiol. Rev..

[48] Bogoyevitch M.A. (2006). The isoform-specific functions of the c-Jun N-terminal Kinases (JNKs): Differences revealed by gene targeting. Bioessays.

[49] Kass R.S., Tsien R.W. (1982). Fluctuations in membrane current driven by intracellular calcium in cardiac Purkinje fibers. Biophys. J..

[50] Anderson M.E., Brown J.H., Bers D.M. (2011). CaMKII in myocardial hypertrophy and heart failure. J. Mol. Cell. Cardiol..

[51] Burstein B., Nattel S. (2008). Atrial structural remodeling as an antiarrhythmic target. J. Cardiovasc. Pharmacol..

[52] Fujimura O., Klein G.J., Yee R., Sharma A.D. (1990). Mode of onset of atrial fibrillation in the Wolff-Parkinson-White syndrome: How important is the accessory pathway?. J. Am. Coll. Cardiol..

